# Maternal TSH and FT4 changes during pregnancy as risk factors for preeclampsia in euthyroid women

**DOI:** 10.3389/fendo.2026.1782499

**Published:** 2026-05-26

**Authors:** Jianxia Lin, Yili Zhang, Yu Meng, Yan Su, Mengfan Song

**Affiliations:** 1 Department of Obstetrics and Gynecology, International Peace Maternity and Child Health Hospital, School of Medicine, Shanghai Jiao Tong University, Shanghai, China; 2 Shanghai Key Laboratory of Embryo Original Diseases, Shanghai, China

**Keywords:** FT4, preeclampsia, pregnancy, thyroid dynamics, thyroid function, TSH

## Abstract

**Objective:**

This large retrospective study aims to investigate the associations between FT4/TSH changes during pregnancy and the risk of preeclampsia among women with normal thyroid function in early and late pregnancy.

**Methods:**

We investigated the associations between changes in FT4 and TSH levels from early to late pregnancy and the risk of preeclampsia using restricted cubic spline and logistic regression models, both unadjusted and adjusted for potential confounders. Threshold effects were assessed via segmented regression to identify possible inflection points. Participants were categorized according to changes in thyroid function, and preeclampsia incidence was compared across the four groups. Sensitivity analyses were performed to evaluate the robustness of the findings. Statistical significance was set at two-sided P < 0.05.

**Results:**

TSH changes showed a J-shaped nonlinear association with preeclampsia risk (threshold 0.68 mIU/L), whereas FT4 changes were linearly and negatively associated. Participants were classified into four thyroid-function groups, with the Complete Decompensation group (ΔTSH > 0.68, ΔFT4 ≤ -3.30) showing the highest risk of preeclampsia (2.7%, aOR = 1.89, P < 0.001) and preeclampsia-related complications, including preterm birth (20.3%, aOR = 3.35, P = 0.016) and low birth weight (18.9%, aOR = 2.46, P = 0.046). Population-attributable risk analysis indicated that 21.6% of preeclampsia cases and 36.6% of severe cases were attributable to this pattern. Sensitivity analyses confirmed the robustness of these findings.

**Conclusion:**

Significant increases in TSH combined with decreases in FT4 during pregnancy are associated with higher risk of preeclampsia and related complications, even among women with normal thyroid function.

## Introduction

During pregnancy, maternal thyroid hormone levels undergo physiological adjustments that are essential for maintaining both maternal and fetal metabolic balance, as well as normal fetal neurodevelopment (1). Previous studies have shown that overt thyroid dysfunction, such as clinical hypothyroidism or hyperthyroidism, is an independent risk factor for adverse pregnancy outcomes including preeclampsia, miscarriage, and preterm birth (2, 3). However, for the large proportion of pregnant women whose thyroid function remains within the normal reference range, it remains unclear whether the longitudinal change patterns of thyroid hormone levels, rather than single-point measurements, represent a more precise and under-recognized dimension of risk stratification. The main focus of this study is to determine whether there are significant individual differences in the adaptability of the maternal thyroid system during pregnancy and whether these differences are reflected in specific patterns of change that are associated with adverse pregnancy outcomes. Among these outcomes, preeclampsia, as a typical placental-origin disease, is of particular interest in relation to thyroid function (4, 5). Therefore, this study aims to systematically evaluate the patterns of maternal thyroid function changes under physiological stress during pregnancy and to explore the association of these changes with the risk of preeclampsia, providing new insights into its potential biological mechanisms and early risk identification.

## Methods

### Study participants

This study is a retrospective cohort study based on electronic medical records from a tertiary maternity hospital in Shanghai, covering the period from May 2013 to December 2016. The study population consisted of pregnant women who underwent systematic prenatal examinations and delivered at the hospital during this period. To clarify the association between patterns of thyroid function changes and the risk of preeclampsia, the study population was selected according to predefined criteria. Inclusion criteria were (1) Chinese nationality, (2) singleton pregnancy, (3) completion of thyroid function tests in early pregnancy (11–13 weeks) and late pregnancy (32–34 weeks), (4) delivery at the study hospital with complete medical records, and (5) postpartum follow-up of more than 12 weeks. Exclusion criteria were (1) multiple pregnancies, (2) pre-existing hypertension or thyroid disease before pregnancy, (3) use of medications affecting thyroid function, (4) presence of other major systemic diseases, (5) non-Chinese nationality, and (6) delivery at another hospital or incomplete/missing information. The study protocol was approved by the Ethics Committee of the hospital (Approval No. GKLW 2019-16). Given that the research involved secondary analysis of anonymized routine clinical data, informed consent was not required. All procedures were conducted in accordance with institutional and ethical guidelines.

### Data collection and laboratory analysis

For all participants, blood samples were collected from the antecubital vein in the morning after an overnight fast during early pregnancy (11–13 weeks) and late pregnancy (32–34 weeks). Serum was separated by centrifugation within six hours of collection. Serum thyroid-stimulating hormone, free thyroxine, and thyroid peroxidase antibody levels were measured quantitatively using the Architect i2000 immunoassay analyzer, with all procedures strictly following the manufacturer’s instructions. TPOAb positivity was defined as a concentration of ≥5.61 IU/mL. Maternal age, parity, education level, *in vitro* fertilization, and other demographic and clinical baseline information were collected through structured interviews at the first prenatal visit. Height and weight were measured directly at the visit and used to calculate pre-pregnancy body mass index (BMI).

### Definition and classification criteria

The definitions and classification criteria applied in this study were based on established medical guidelines and relevant literature. 1) Normal reference ranges for TSH and FT4 were defined according to current international guidelines, which primarily advocate the use of laboratory- and trimester-specific reference intervals, as general population values are not applicable to pregnant women (6). In the present study, trimester-specific reference ranges were determined for early pregnancy (11–13 weeks) and late pregnancy (32–34 weeks) by calculating the 2.5th and 97.5th percentiles of TSH and FT4. The resulting normal ranges for the first trimester were 0.03–3.64 mIU/L for TSH and 11.7–19.6 pmol/L for FT4, whereas the ranges for the third trimester were 0.39–3.67 mIU/L for TSH and 9.1–14.4 pmol/L for FT4. 2) Preeclampsia: According to ACOG guidelines (7), preeclampsia is defined as systolic blood pressure ≥140 mmHg or diastolic blood pressure ≥90 mmHg on two occasions at least 4 hours apart after 20 weeks of gestation in a previously normotensive woman, along with either 24-hour urinary protein excretion ≥300 mg or a protein/creatinine ratio ≥0.3. 3) Overweight/obesity Definition: BMI was calculated as weight in kilograms divided by height in meters squared (kg/m²). Overweight/obesity was defined as a BMI ≥ 25 kg/m², according to the pre-pregnancy body mass index cut points for Asian populations recommended in the literature (8, 9).

### Statistical analysis

To examine the nonlinear relationship between changes in FT4 and TSH levels from early to late pregnancy and the risk of preeclampsia, we used restricted cubic spline (RCS) models. The knots for the RCS models were automatically selected based on the Akaike Information Criterion (AIC) to optimize model fit and complexity. RCS models were fitted both unadjusted and adjusted for potential confounders. P-values for both overall and nonlinear associations were calculated for each model. Threshold effect analysis was conducted using the “segmented” package in R. First, standard linear regression (Model 1) was used to assess the overall association between changes in TSH and FT4 levels and the risk of preeclampsia. Subsequently, a two-segment linear regression model (Model 2) was constructed to identify the inflection point for TSH changes and assess whether a threshold effect exists between TSH changes and preeclampsia, using a likelihood ratio test to evaluate model fit. Sensitivity analyses were performed to further confirm the independent and robust association between thyroid function changes during pregnancy and the risk of preeclampsia. Statistical significance was set at two-sided P < 0.05.

## Result

### Participants

**Figure 1:**
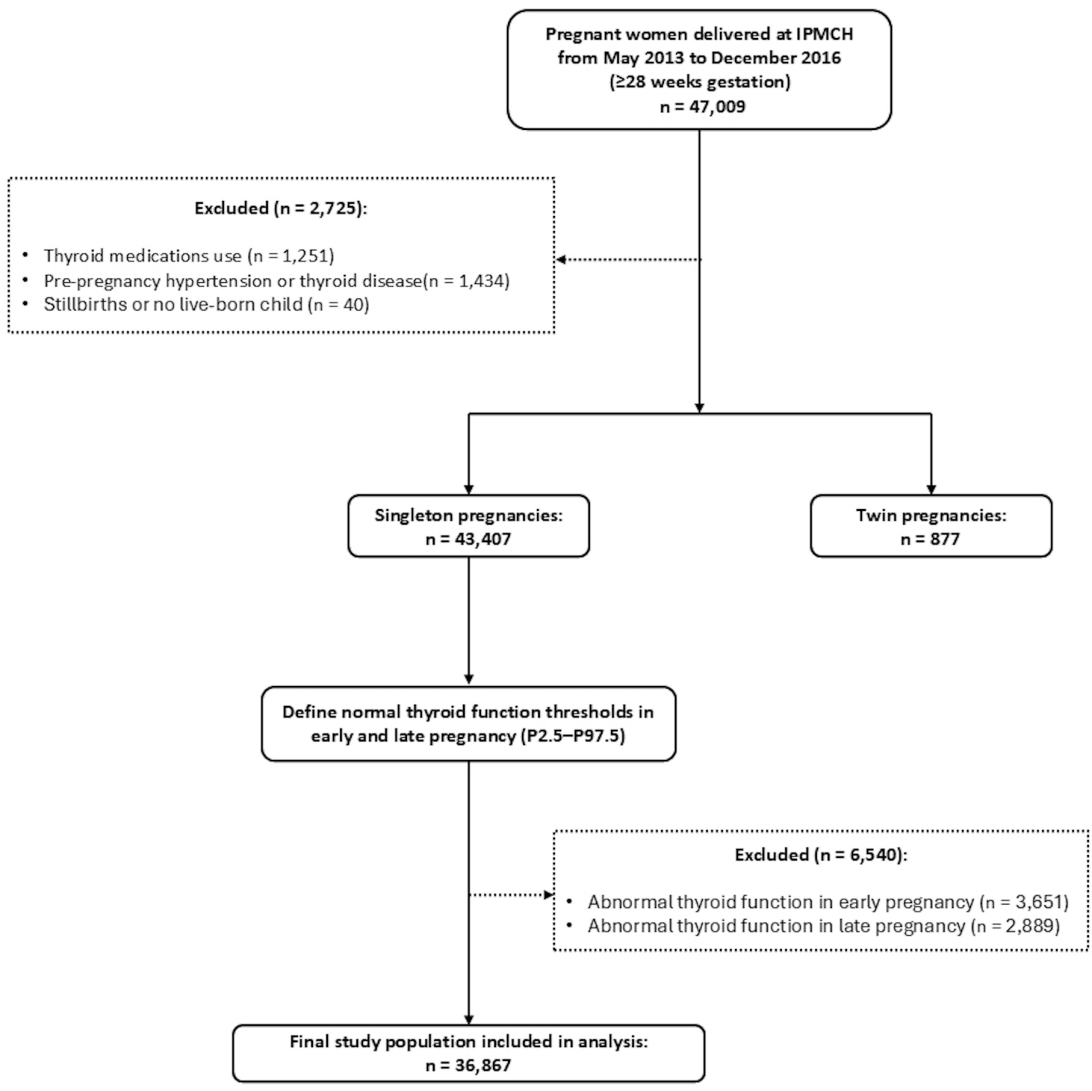
Study population selection flow for singleton pregnancies with normal thyroid function.

For this retrospective analysis, a total of 47,009 women who delivered at a tertiary specialized hospital between May 2013 and December 2016 were identified. Based on the exclusion criteria, the following cases were excluded: use of thyroid medications (n=1,251), pre-pregnancy hypertension or thyroid disease (n=1,434), stillbirth or no live-born child (n=40), and twin pregnancies (n=877). The remaining cohort included 43,407 women with singleton pregnancies. Normal thyroid function thresholds for early and late pregnancy were defined using the P2.5-P97.5 range. The normal ranges for the first trimester were 0.03–3.64 mIU/L for TSH and 11.7–19.6 pmol/L for FT4, while the third trimester ranges were 0.39–3.67 mIU/L for TSH and 9.1–14.4 pmol/L for FT4. A total of 3,651 women were excluded for abnormal thyroid function in early pregnancy, and 2,889 for abnormal thyroid function in late pregnancy. After excluding these 6,540 individuals, the final study population consisted of 36,867 women with singleton pregnancies. The entire inclusion and exclusion process is shown in **Figure 1**.

### Association between changes in TSH/FT4 levels and preeclampsia risk

**Figure 2:**
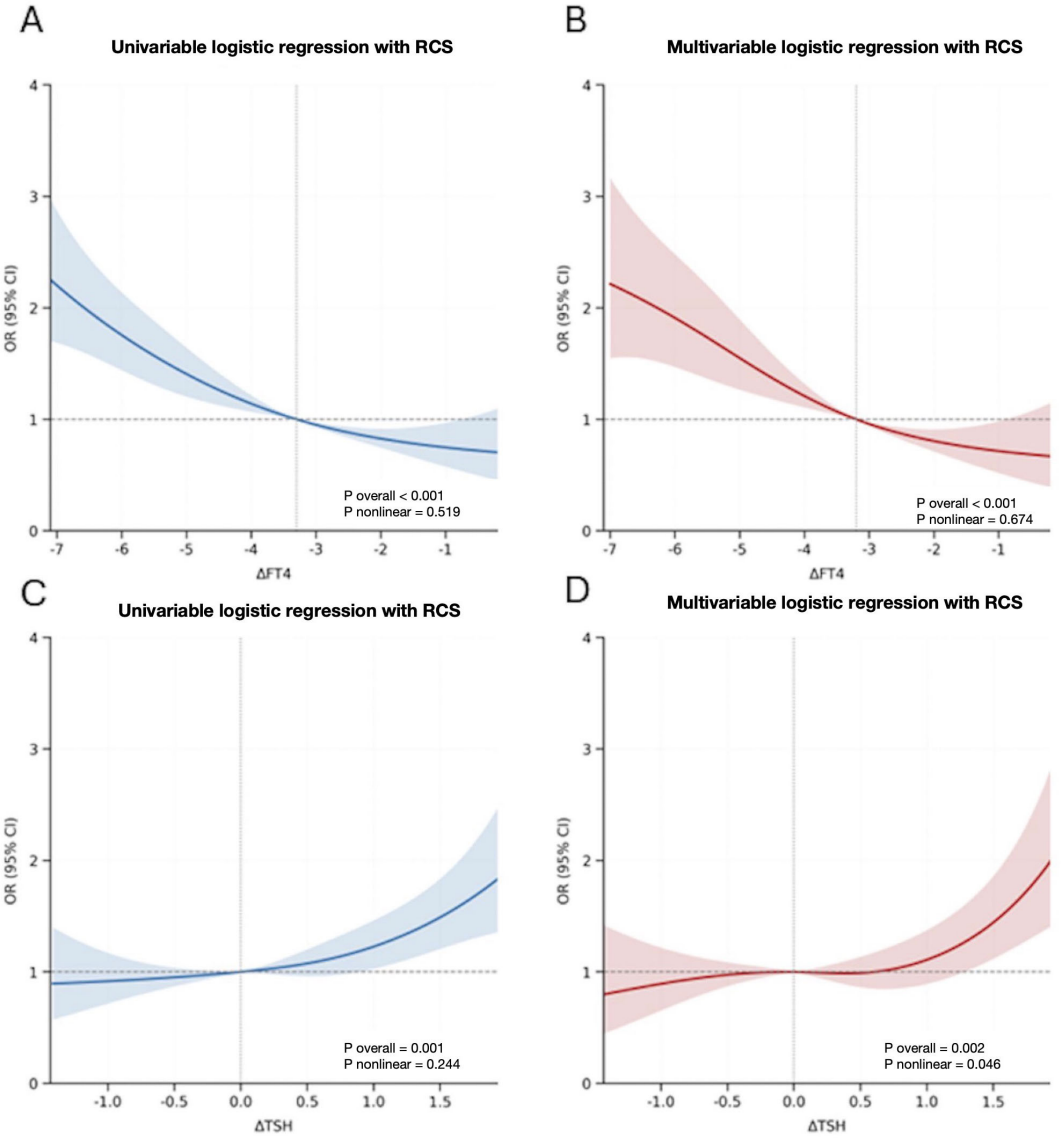
Restricted cubic spline (RCS) curves illustrating the association between changes in FT4/TSH and the risk of preeclampsia. Panels **(A, B)** depict the relationship between changes in FT4 from early to late pregnancy and the odds ratio (OR) for preeclampsia, whereas Panels **(C, D)** illustrate the corresponding associations for changes in TSH. Solid lines represent estimated ORs, and shaded areas indicate 95% confidence intervals (CIs). Knots for the RCS models were selected automatically based on the Akaike Information Criterion (AIC) to optimize model fit and complexity. Panels **(A, C)** (blue) show unadjusted estimates, whereas Panels **(B, D)** (red) present estimates adjusted for maternal age, pre-pregnancy BMI, parity, education level, IVF, TPOAb status, smoking, alcohol consumption, and GDM. P-values for overall and nonlinear associations are reported in each panel. The wider CIs at the extreme FT4/TSH change values likely reflect sparse data in these regions and should therefore be interpreted with caution.

The relationship between maternal FT4/TSH change levels and the risk of preeclampsia was analyzed using RCS models combined with univariable and multivariable logistic regression analysis (as shown in **Figure 2**). The multivariable models were adjusted for potential confounders, including maternal age, pre-pregnancy BMI, parity, education level, IVF, TPOAb status, smoking, alcohol consumption, and GDM. RCS regression showed a J-shaped nonlinear association between TSH change levels and preeclampsia risk (P for overall = 0.002, P for nonlinearity = 0.046). In contrast, FT4 change levels were negatively linearly associated with preeclampsia risk (P for overall < 0.001, P for nonlinearity = 0.674). Subsequently, threshold effect analysis identified 0.68 as the key inflection point for TSH change levels, which was used in segmented regression to further clarify the relationship with preeclampsia risk. Segmented regression analysis revealed that when TSH change < 0.68, the association was not significant [OR (95% CI): 0.83 (0.62–1.12), P = 0.231], whereas when TSH change ≥ 0.68, a significant positive association with preeclampsia risk was observed [OR (95% CI): 1.84 (1.24–2.73), P = 0.002]. Likelihood ratio testing confirmed that the segmented regression model fit the data better than the linear model (P = 0.034), supporting the presence of a threshold effect at a TSH change level of 0.68. Although FT4 change was negatively associated with preeclampsia risk overall [OR (95% CI): 0.65 (0.59–0.72), P < 0.001], segmented linear regression analysis did not reveal a significant threshold effect (P for likelihood test = 0.264), suggesting that there was no clear threshold effect in the association between FT4 change and preeclampsia.

### Classification based on thyroid function changes during pregnancy

**Figure 3:**
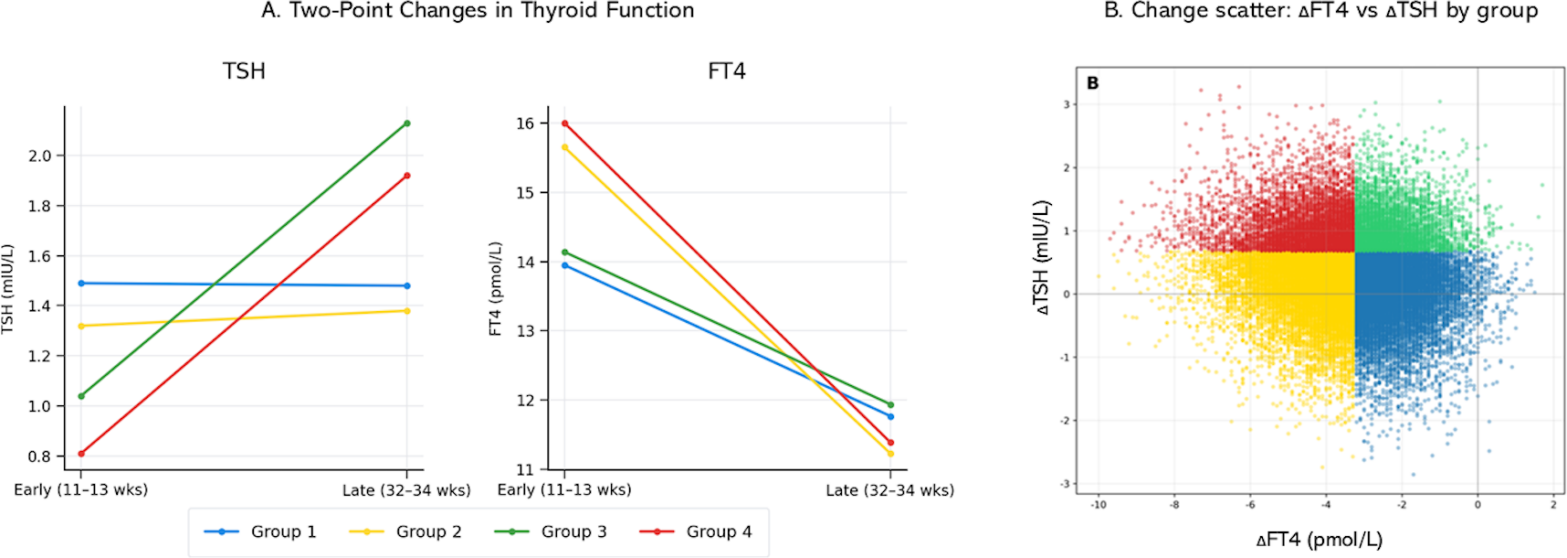
Combined visualization of thyroid function dynamics and change scatter by group. **(A)** Two-Point Changes in Thyroid Function: Lines represent mean absolute changes from early pregnancy (11–13 weeks) to late pregnancy (32–34 weeks) for each group: Error bars indicate 95% confidence intervals at the late pregnancy time point. Colors distinguish groups (Group 1: blue, Group 2: yellow, Group 3: green, Group 4: red). Units: TSH (mIU/L) and FT4 (pmol/L) are on the Y-axis. **(B)** Bivariate change scatter (ΔFT4 vs ΔTSH) for individual subjects, colored by group: Horizontal and vertical gray lines indicate zero change, dividing the plot into quadrants representing increase or decrease in each analyte.

In this study, participants were classified based on changes in thyroid function during pregnancy. For TSH change, the clinical threshold identified by threshold effect analysis was 0.68 mIU/L, dividing TSH change into two groups: > 0.68 mIU/L and ≤ 0.68 mIU/L. For FT4 change, due to its linear negative association with preeclampsia risk, the median FT4 change value was calculated to be -3.30, dividing FT4 change into two groups: > -3.30 mIU/L and ≤ -3.30 mIU/L. Participants were then divided into four groups based on thyroid function changes during pregnancy, as shown in **Figure 3**. Group 1 (TSH change ≤ 0.68, FT4 change > -3.30) represented the stable ideal type; Group 2 (TSH change ≤ 0.68, FT4 change ≤ -3.30) represented the latent decompensation type; Group 3 (TSH change > 0.68, FT4 change > -3.30) represented the high-tension compensatory type; and Group 4 (TSH change > 0.68, FT4 change ≤ -3.30) represented the complete decompensated type. The baseline characteristics of participants across these four groups are presented in **Table 1**.

**Table 1:** Baseline characteristics of study participants by group.

Variable	Category	Group 1 (N = 14,050)	Group 2 (N = 13,310)	Group 3 (N = 4,088)	Group 4 (N = 5,419)
Parity	Nulliparous	10,912 (77.7%)	11,416 (85.8%)	3,274 (80.1%)	4,714 (87.0%)
	Multiparous	3,138 (22.3%)	1,894 (14.2%)	814 (19.9%)	705 (13.0%)
Education	Bachelor’s degree or above	2,756 (19.6%)	2,370 (17.8%)	718 (17.6%)	926 (17.1%)
	High school or below	11,294 (80.4%)	10,940 (82.2%)	3,370 (82.4%)	4,493 (82.9%)
IVF conception	Yes	380 (2.7%)	389 (2.9%)	140 (3.4%)	146 (2.7%)
	No	13,670 (97.3%)	12,921 (97.1%)	3,948 (96.6%)	5,273 (97.3%)
GDM	Yes	1,613 (11.5%)	1,202 (9.0%)	447 (10.9%)	543 (10.0%)
	No	12,437 (88.5%)	12,108 (91.0%)	3,641 (89.1%)	4,876 (90.0%)
TPOAb	Positive	1,738 (12.4%)	1,255 (9.4%)	248 (6.1%)	301 (5.6%)
	Negative	12,312 (87.6%)	12,055 (90.6%)	3,840 (93.9%)	5,118 (94.4%)
Smoking	Yes	11 (0.1%)	11 (0.1%)	2 (0.1%)	2 (0.0%)
	No	14,039 (99.9%)	13,299 (99.9%)	4,086 (99.9%)	5,417 (100.0%)
Alcohol consumption	Yes	2 (0.0%)	0 (0.0%)	1 (0.0%)	0 (0.0%)
	No	14,048 (100.0%)	13,310 (100.0%)	4,087 (100.0%)	5,419 (100.0%)
Age (years)		30.41 ± 3.67	29.86 ± 3.47	30.20 ± 3.74	29.84 ± 3.50
Gestational age at sampling (weeks)		39.03 ± 1.20	39.08 ± 1.20	38.96 ± 1.25	39.02 ± 1.25
Baseline early-pregnancy FT4 (pmol/L)		13.95 ± 1.15	15.65 ± 1.38	14.14 ± 1.18	16.00 ± 1.43
Baseline early-pregnancy TSH (mIU/L)		1.50 ± 0.75	1.32 ± 0.76	1.04 ± 0.57	0.81 ± 0.56
Pre-pregnancy BMI (kg/m²)		21.24 ± 2.86	20.89 ± 2.56	21.05 ± 2.85	20.75 ± 2.74

Values are presented as n (%) for categorical variables and mean ± standard deviation (SD) for continuous variables. IVF, in vitro fertilization; GDM, gestational diabetes mellitus; TPOAb, thyroid peroxidase antibody. No statistical comparisons were performed across groups, since all variables will be adjusted in subsequent multivariable analyses.

### Comparison of preeclampsia incidence across the four groups

Using Group 1 (TSH change ≤ 0.68, FT4 change > -3.30, Stable Ideal) as the reference group, we compared the incidence of preeclampsia across the four groups. The findings presented in **Table 2** indicated that the overall preeclampsia incidence in the study population was 1.9% (684/36,867). Group 4 (TSH change > 0.68, FT4 change ≤ -3.30, Complete Decompensation) had the highest preeclampsia incidence (2.7%), with an adjusted OR of 1.89 (95% CI: 1.44–2.48, P < 0.001). Group 2 (TSH change ≤ 0.68, FT4 change ≤ -3.30, Latent Decompensation) and Group 3 (TSH change > 0.68, FT4 change > -3.30, High-Tension Compensation) had moderate preeclampsia incidence (2.0% and 1.6%), with adjusted ORs of 1.48 (95% CI: 1.18–1.85, P < 0.001) and 1.08 (95% CI: 0.77–1.52, P = 0.653), respectively. In contrast, Group 1 (TSH change ≤ 0.68, FT4 change > -3.30) had the lowest preeclampsia incidence (1.5%). These findings suggest that the highest risk of preeclampsia occurs in Group 4 when both TSH increases significantly (> 0.68 mIU/L) and FT4 decreases significantly (≤ -3.30 pmol/L), while individual increases in TSH or decreases in FT4 result in moderate risk, and stable TSH and FT4 levels (Group 1) are associated with the lowest risk.

**Table 2:** Association between grouped two-point change patterns in thyroid function and preeclampsia risk.

Group	N	Preeclampsia incidence (%)	Clinical features			Univariate analysis		Multivariate analysis	
			TSH change (mIU/L)	FT4 change (pmol/L)	Pattern Type	OR (95%CI)	P	OR (95%CI)	P
1	14,050	208 (1.5%)	≤ 0.68	> -3.30	Stable Ideal	Reference		Reference	
2	13,310	262 (2.0%)	≤ 0.68	≤ -3.30	Latent Decompensation	1.34 (1.11 ~ 1.61)	0.002*	1.48 (1.18 ~ 1.85)	<0.001*
3	4,088	66 (1.6%)	> 0.68	> -3.30	High-Tension Compensation	1.09 (0.83 ~ 1.44)	0.536	1.08 (0.77 ~ 1.52)	0.653
4	5,419	148 (2.7%)	> 0.68	≤ -3.30	Complete Decompensation	1.87 (1.51 ~ 2.31)	<0.001*	1.89 (1.44 ~ 2.48)	<0.001*

Groups were created based on statistically meaningful cutoffs. Univariate analysis was calculated using logistic regression without adjustment. Multivariate analysis was adjusted for maternal age, pre-pregnancy BMI, parity, education level, IVF, TPOAb status, smoking, alcohol consumption, GDM and early-onset preeclampsia. Group 1 served as the reference group. Abbreviations: OR, odds ratio; CI, confidence interval; TSH, thyroid-stimulating hormone; FT4, free thyroxine. * indicates P < 0.05.

### Association of four groups with preeclampsia complicated by preterm birth and low birth weight

Preterm birth and low birth weight (LBW) were used as substitute markers of placental dysfunction to reflect the risk of placental type preeclampsia (10), which has a poorer prognosis than the maternal type (11). Early differentiation of potential preeclampsia patients into placental or maternal types is considered crucial, as management strategies differ between the two (12). Therefore, we further investigate the relationship between change patterns in maternal thyroid function and the risk of preeclampsia with complications, such as preterm birth and LBW. A subgroup analysis of preeclampsia patients was conducted based on the occurrence of preterm birth (<37 weeks) or LBW (<2500 g). The associations between the four subgroups and these outcomes were assessed using both univariate and multivariate logistic regression analyses, adjusting for maternal age, pre-pregnancy BMI, parity, education level, IVF status, TPOAb status, smoking, alcohol consumption, GDM, and early-onset preeclampsia. In preeclampsia patients, different patterns of thyroid function changes were closely associated with the occurrence of preterm birth and LBW (**Table 3**). For preterm birth, multivariate analysis showed that women in the Complete Decompensation group (Group 4) had a significantly increased risk (aOR = 3.35, 95% CI: 1.26–8.91, P = 0.016). The High-Tension Compensation group (Group 3) showed an increased trend in risk, but the result was marginally significant (aOR = 3.01, 95% CI: 0.98–9.27, P = 0.054), while the Latent Decompensation group (Group 2) did not show a significant association with preterm birth (P = 0.200). For LBW, women in the Complete Decompensation group (Group 4) also had a significantly increased risk (aOR = 2.46, 95% CI: 1.02–5.96, P = 0.046). Notably, the High-Tension Compensation group (Group 3) also showed a significant increase in risk (aOR = 3.18, 95% CI: 1.13–9.00, P = 0.029), while the Latent Decompensation group (Group 2) showed an increase in risk, but it did not reach statistical significance. Overall, when pregnant women exhibited a significant increase in TSH levels along with a decrease in FT4 (Complete Decompensation), they were more likely to experience adverse outcomes such as preterm birth and LBW. This suggests that this thyroid function change pattern may be associated with the occurrence and severity of preeclampsia.

**Table 3:** Association of group with preeclampsia complicated by preterm birth and low birth weight.

Group	Preterm birth among preeclampsia patients					Low birth weight among preeclampsia patients				
	n/N (%)	Univariate analysis		Multivariate analysis		n/N (%)	Univariate analysis		Multivariate analysis	
		OR (95% CI)	P	aOR (95% CI)	P		OR (95% CI)	P	aOR (95% CI)	P
1	16/208 (7.7%)	Reference				13/208 (6.3%)	Reference			
2	21/262 (8.0%)	1.05 (0.53–2.06)	0.897	1.86 (0.72, 4.83)	0.200	18/262 (6.9%)	1.11 (0.53–2.31)	0.788	1.38 (0.58, 3.29)	0.467
3	12/66 (18.2%)	2.67 (1.19–5.98)	0.017*	3.01 (0.98, 9.27)	0.054	13/66 (19.7%)	3.68 (1.61–8.41)	0.002*	3.18 (1.13, 9.00)	0.029*
4	30/148 (20.3%)	3.05 (1.59–5.84)	<0.001*	3.35 (1.26, 8.91)	0.016*	28/148 (18.9%)	3.50 (1.75–7.02)	<0.001*	2.46 (1.02, 5.96)	0.046*

N represents the number of preeclampsia patients in each group, n represents the number of preeclampsia patients with the outcome, and % represents the corresponding percentage (n/N). Univariate ORs were calculated using logistic regression without adjustment. Multivariate analysis was adjusted for maternal age, pre-pregnancy BMI, parity, education level, IVF, TPOAb status, smoking, alcohol consumption, GDM and early-onset preeclampsia. Group 1 served as the reference group. Abbreviations: OR, odds ratio; CI, confidence interval; LBW, low birth weight.

### Population attributable risk

We further estimated the population-attributable risk (PAR) associated with the thyroid “Complete Decompensation” state. Population attributable risk (PAR%) was calculated to estimate the proportion of preeclampsia cases in the study population theoretically attributable to thyroid complete decompensation using the formula: $\text{PAR}\%=\frac{Pe\times (RR-1)}{Pe\times (RR-1)+1}\times 100\%$, where Pe is the proportion of exposed individuals, and RR is approximated by OR given the low incidence of preeclampsia. In the overall cohort, approximately 21.6% of preeclampsia cases could be attributed to this thyroid dysfunction. Among patients with severe composite adverse outcomes, defined as preeclampsia complicated by preterm birth or low birth weight, the attributable proportion rose to 36.6%, indicating a stronger contribution of thyroid decompensation to severe preeclampsia phenotypes. These results emphasize the clinical and public health significance of the thyroid “Complete Decompensation” state. Effective prevention or management of this condition could theoretically prevent around one-fifth of all preeclampsia cases and over one-third of cases with severe adverse outcomes. This highlights the importance of systematic monitoring of maternal thyroid function during pregnancy, as timely identification and intervention for abnormal patterns of thyroid function changes may offer a promising strategy to reduce both the incidence and severity of preeclampsia. It should be noted that OR overestimates RR, and thus, the true PAR may be slightly lower. Additionally, PAR assumes a causal relationship between exposure and outcome. Given the observational design of this study, the PAR estimates should be interpreted as hypothetical and not as evidence of causality.

### Sensitivity analyses

To assess the robustness of our findings, we conducted sensitivity analyses comparing the lowest-risk (Group 1, Stable Ideal) and highest-risk (Group 4, Complete Decompensation) thyroid function patterns by sequentially excluding specific subpopulations, including multiparous women, IVF pregnancies, overweight/obese women (BMI ≥25 kg/m²), advanced maternal age (≥35 years), TPOAb-positive women, and cases of early-onset preeclampsia (**Table 4**). In the sensitivity analysis, we examined the impact of different definitions of obesity on the study results using two separate BMI cutoffs. In addition to applying the Asian criteria (8, 9), where BMI ≥25 kg/m² is defined as overweight/obesity, we also used the WHO international cutoff (13), where BMI ≥30 kg/m² is defined as obesity, to assess the robustness of the study findings under a more widely accepted global standard. In all analyses, women in the Complete Decompensation group consistently showed significantly higher preeclampsia risk compared with the Stable Ideal group (P < 0.001), confirming that the association between significant increases in TSH combined with decreases in FT4 and preeclampsia is robust and independent of maternal characteristics or high-risk factors. Clinically, this underscores the importance of continuous monitoring of maternal thyroid function changes. Even among women with thyroid levels within the normal range, attention should be paid to the Complete Decompensation pattern to identify potential high-risk pregnancies early and optimize management strategies.

**Table 4:** Sensitivity analysis comparing Group 1 and Group 4 thyroid function patterns by excluding specific subpopulations.

Variable	Subgroup total (n, %)	Preeclampsia incidence		OR (95% CI)	P
		Group 1	Group 4		
Total (Group 1 and Group 4)	19,469 (100.0%)	1.5%	2.7%	1.91 (1.45 ~ 2.52)	<0.001
Excluding multiparous women	15,626 (80.26%)	1.7%	3.0%	1.95 (1.46 ~ 2.60)	<0.001
Excluding IVF conceptions	18,943 (97.30%)	1.4%	2.7%	1.98 (1.49 ~ 2.61)	<0.001
Excluding overweight/obese individuals (BMI ≥25 kg/m²)	17,793 (91.39%)	1.3%	2.6%	1.88 (1.39 ~ 2.55)	<0.001
Excluding obese individuals (BMI ≥ 30 kg/m²)	19,264 (98.95%)	1.5%	2.9%	1.81 (1.37 ~ 2.39)	<0.001
Excluding advanced age (≥35 years)	16,967 (87.15%)	1.4%	2.7%	2.11 (1.57 ~ 2.84)	<0.001
Excluding TPOAb positive individuals	17,430 (89.53%)	1.5%	2.6%	1.80 (1.35 ~ 2.39)	<0.001
Excluding high school or below education	15,182 (77.98%)	1.4%	3.0%	2.21 (1.62 ~ 3.01)	<0.001
Excluding early-onset PE	19,451 (99.91%)	1.4%	2.5%	1.91 (1.45 ~ 2.52)	<0.001
Excluding GDM	17,313 (88.93%)	1.4%	2.6%	1.98 (1.47 ~ 2.66)	<0.001
Excluding smoking	19,456 (99.93%)	1.5%	2.7%	1.91 (1.45 ~ 2.52)	<0.001
Excluding alcohol consumption	19,467 (99.99%)	1.5%	2.7%	1.91 (1.45 ~ 2.52)	<0.001

This table presents sensitivity analyses comparing the lowest-risk (Group 1) and highest-risk (Group 4) thyroid function patterns. Incidences were calculated based on the original data (cases/total in the subgroup). All ORs were derived from logistic regression models adjusted for maternal age, pre-pregnancy BMI, parity, education level, IVF, TPOAb status, smoking, alcohol consumption, GDM and early-onset PE, unless the variable itself was the exclusion criterion. BMI, body mass index; CI, confidence interval; IVF, in vitro fertilization; OR, odds ratio; TPOAb, thyroid peroxidase antibody, GDM, gestational diabetes mellitus; PE, preeclampsia.

## Discussion

During pregnancy, maternal thyroid hormone levels undergo physiological adjustments to meet the increasing metabolic demands of both mother and fetus and to maintain energy homeostasis (14). While previous studies have emphasized the importance of dynamic monitoring of thyroid function during gestation (15), evidence linking longitudinal thyroid changes to complications such as preeclampsia remains limited.

Previous studies showed that trophoblasts express thyroid hormone receptors and respond to TH signaling, promoting trophoblast invasion, angiogenesis, and the expression of key molecules required for proper placental development (16). Low maternal FT4 may cause metabolic dysregulation, promote inflammation and immune imbalance at the maternal–fetal interface, and impair endothelial function through oxidative stress, thereby potentially contributing to the development of preeclampsia (17–19). Abnormal placental expression of TH transporters, deiodinases, and receptors is also associated with preeclampsia and fetal growth restriction (16). Animal studies further indicate that gestational hypothyroxinemia upregulates placental pro-inflammatory cytokines such as IL-6 and IL-17, promotes M1 macrophage polarization, and impairs trophoblast function (17). Elevated TSH, as a peripheral compensatory marker of relative thyroid hormone deficiency, may affect placental function through multiple pathways, increasing the risk of preeclampsia (20). Clinical studies have also reported positive associations between elevated TSH and preeclampsia, fetal growth restriction, and low birth weight (21). After excluding central disorders, TSH is considered the most sensitive and convenient measure of thyroid functional status (22). Compared with peripheral hormones, its elevation signals compensatory or early decompensatory states. TSH may also play a direct role in placental physiology. Placental and decidual tissues express TSH receptors, and TSH signaling regulates trophoblast proliferation, differentiation, invasion, and angiogenesis (23). Maternal thyroid signaling disruption is commonly observed in preeclampsia and other placental disorders (16), and abnormal invasive placentation has been associated with altered TSH levels, suggesting a link to placental vascular remodeling (24). Therefore, the elevated TSH patterns we observed may reflect both thyroid compensation and local placental stress, indicating that a persistent rise in TSH, even within the reference range, may serve as an early warning of impaired placental perfusion and increased preeclampsia risk.

Most previous studies have relied on thyroid function measurements at a single time point during pregnancy or compared average levels across pregnant women using cross-sectional data. Such static analyses have highlighted associations between abnormal FT4 or TSH levels and the risk of preeclampsia, providing preliminary clinical insights. However, the HPT feedback system is dynamic and adaptive (25). A single static measurement cannot capture its full trajectory and misses critical dynamic information about the transition from normal adaptation to decompensation, making it difficult to identify high-risk evolution patterns. In addition, cross-sectional studies cannot reflect the temporal interactions between TSH and FT4 or the compensatory mechanisms over time. Therefore, this study proposes that clinical evaluation of thyroid function during pregnancy should shift from traditional “point-in-time diagnosis” to “two-point based classification.” By assessing the two-point changes of TSH and FT4 and their combined patterns, we systematically highlighted their association with preeclampsia risk, addressing a gap in current research. Furthermore, by grouping women based on the magnitude of changes in TSH and FT4, we preliminarily defined four change subtypes, allowing identification of patterns such as “high-tension compensation” and “complete decompensation.” This approach not only reveals the temporal relationship between dynamic changes in TSH and FT4 and preeclampsia risk but also indicates that two-point changes may be more sensitive than single-point values for detecting thyroid decompensation and placental dysfunction. This trajectory-based framework provides a theoretical basis for precise risk stratification and mechanism-specific prevention of preeclampsia. Previous studies have applied latent class growth analysis (LCGA) to longitudinal TSH and FT4 data (26, 27). These studies demonstrated that LCGA can classify pregnant women according to individual longitudinal thyroid hormone trajectories and assess the predictive value of each trajectory for adverse outcomes. However, the clinical application of these models remains challenging due to the need for multiple follow-up measurements and complex computational analysis. In contrast, our study proposes a simpler classification method based on clinically meaningful thresholds of ΔTSH and ΔFT4, allowing early pregnancy risk stratification and providing practical guidance for clinical decision-making.

This study is the first to systematically evaluate the relationship between two-point changes in thyroid function during pregnancy and the risk of preeclampsia. Our findings suggest that even among euthyroid women in early pregnancy, changes in FT4 and TSH can independently predict the risk of preeclampsia. Based on the combined changes in TSH and FT4, we proposed four distinct thyroid function change patterns, providing a practical tool for identifying women at high risk for preeclampsia. A significant increase in TSH marks a high-compensation state, and once it exceeds a critical threshold (such as 0.68 mIU/L in this study), it significantly elevates the risk of preeclampsia. In contrast, a sustained decline in FT4 reflects thyroid dysfunction, which continuously raises the risk of preeclampsia. When both TSH and FT4 undergo significant changes simultaneously, this signals complete thyroid axis decompensation, particularly in pregnant women with preeclampsia, where the risk of complications such as preterm birth and low birth weight is increased. These findings highlight the importance of thyroid function change patterns during pregnancy and advocate for a shift from traditional static assessments to dynamic monitoring strategies based on relative changes. This approach facilitates clinical risk stratification and early intervention. Future research should validate the predictive value of this dynamic trajectory and explore whether individualized interventions (such as levothyroxine supplementation or targeted modulation of specific metabolic and immune pathways) can improve placental function and reduce the incidence of preeclampsia.

### Strengths and limitations

The key strength of this study is its large sample size and its novel focus on thyroid function changes, as well as the potential clinical applicability of the findings. Unlike previous research that relied on single time-point assessments, we proposed, for the first time, a two points based classification method using changes in TSH and FT4 from early to late pregnancy, enabling intuitive differentiation of maternal thyroid function status. This classification framework is easy to implement. It is especially useful for identifying women whose thyroid function appears normal in both early and late pregnancy but shows high-risk trends over time, enabling earlier risk stratification and guiding targeted monitoring or preventive interventions. Furthermore, rigorous statistical methods were employed, including data-driven threshold effect analysis to determine cut-off values, multiple sensitivity analyses to validate the robustness of the classification. However, several limitations regarding potential confounders should be considered. First, while we adjusted for major factors including maternal age, pre-pregnancy BMI, parity, GDM, smoking status, alcohol consumption, and early-onset preeclampsia, residual confounding may still remain due to the retrospective design. Specifically, individual iodine nutritional status (e.g., urinary iodine concentration) and exact gestational age at blood sampling were not available, which could influence thyroid hormone measurements. Although our cohort is from an iodine-sufficient region (28), individual variations in iodine intake may still exist. Even within the narrow windows of 11–13 and 32–34 weeks, slight differences in gestational age at sampling may affect hormone levels. In addition, we were unable to adjust for gestational weight gain; however, pre-pregnancy BMI was included as a covariate to partially account for baseline metabolic status. Future prospective studies with more comprehensive phenotypic and longitudinal data are warranted to further validate our findings. Finally, this study involved multiple group comparisons across several outcomes. Therefore, the possibility of inflated type I error cannot be fully excluded, particularly for associations with marginal statistical significance. These results should nevertheless be interpreted with appropriate caution. Considering that the pathophysiology of preeclampsia may itself affect thyroid function, we adjusted for early-onset preeclampsia in multivariable analyses and performed sensitivity analyses excluding these cases. However, as this was an observational study, reverse causality cannot be completely ruled out, and the observed associations should be interpreted as indicative rather than causal.

## Data Availability

The original contributions presented in the study are included in the article/**Supplementary Material**. Further inquiries can be directed to the corresponding authors.
