# Supplementary material for: Maternal TSH and FT4 changes during pregnancy as risk factors for preeclampsia in euthyroid women
Source: Front Endocrinol (Lausanne). 2026 May 26;17:1782499. doi: 10.3389/fendo.2026.1782499 (PMC13246337; doi:10.3389/fendo.2026.1782499)
Supplement: Supplementary file 1 [file DataSheet1.docx]

# ---------------------------

# RCS-logit panel plot (linear y) from "RCS data.xlsx"

# ---------------------------

# Packages

library(readxl)

library(dplyr)

library(tidyr)

library(ggplot2)

library(splines)

library(purrr)

# ---- 1) Load data

file_path <- "RCS data.xlsx"

df <- readxl::read_excel(path = file_path)

# Basic column checks

required <- c("pre_ecl", "FT4_change", "TSH_change")

stopifnot(all(required %in% names(df)))

# Outcome 0/1

df <- df %>%

  mutate(pre_ecl = as.integer(pre_ecl > 0))

# ---- 2) Select covariates

all_cols <- names(df)

last10 <- setdiff(tail(all_cols, 10), c("pre_ecl", "FT4_change", "TSH_change"))

if (length(last10) < 10) {

  # backfill from the end if needed

  extras <- rev(setdiff(rev(all_cols), c("pre_ecl", "FT4_change", "TSH_change", last10)))

  covariates <- unique(c(last10, extras))[seq_len(min(10, length(unique(c(last10, extras)))))]

} else {

  covariates <- last10

}

# Drop earlyonset if present

covariates <- setdiff(covariates, "earlyonset")

# Identify categorical covariates (character or factor)

cat_covs <- covariates[sapply(df[covariates], function(x) is.character(x) || is.factor(x))]

# Helper to build RHS covariate formula

cov_terms <- if (length(covariates) == 0) {

  ""

} else {

  paste0(ifelse(covariates %in% cat_covs,

                paste0("factor(`", covariates, "`)"),

                paste0("`", covariates, "`")),

         collapse = " + ")

}

# ---- 3) Core helpers

# Typical values: median for numeric, mode for categorical

typical_values <- function(data, covs, cat_covs) {

  vals <- list()

  for (c in covs) {

    v <- data[[c]]

    if (c %in% cat_covs) {

      # mode (most frequent non-missing)

      tab <- sort(table(v), decreasing = TRUE)

      vals[[c]] <- names(tab)[1]

    } else {

      vals[[c]] <- stats::median(as.numeric(v), na.rm = TRUE)

    }

  }

  vals

}

# Build model matrix for any dataset under a fitted glm

# and return X(new) - X(ref) to anchor OR at reference

delta_design <- function(fit, newdata, refdata) {

  X_new <- model.matrix(stats::terms(fit), newdata)

  X_ref <- model.matrix(stats::terms(fit), refdata)

  # In case column ordering differs, align by colnames

  common <- intersect(colnames(X_new), colnames(X_ref))

  X_new <- X_new[, common, drop = FALSE]

  X_ref <- X_ref[, common, drop = FALSE]

  X_new - X_ref

}

# Compute OR curve and 95% CI along a grid for a single variable

or_curve <- function(fit, varname, x_grid, ref_value, fixed_vals = NULL) {

  beta <- coef(fit)

  V <- vcov(fit)

  ref_list <- list()

  ref_list[[varname]] <- ref_value

  # Add fixed values

  if (!is.null(fixed_vals)) ref_list <- c(ref_list, fixed_vals)

  ref_df <- as.data.frame(ref_list)

  # Ensure all needed columns exist in newdata/refdata

  # Extract model variables from formula

  vars_in_model <- all.vars(formula(fit))

  for (nm in setdiff(vars_in_model, names(ref_df))) {

    # Try to fill numerics with 0 and factors with first level as a fallback

    if (is.factor(model.frame(fit)[[nm]])) {

      ref_df[[nm]] <- levels(model.frame(fit)[[nm]])[1]

    } else {

      ref_df[[nm]] <- 0

    }

  }

  res <- purrr::map_dfr(x_grid, function(x) {

    new_list <- ref_list

    new_list[[varname]] <- x

    new_df <- as.data.frame(new_list)

    for (nm in setdiff(vars_in_model, names(new_df))) {

      if (is.factor(model.frame(fit)[[nm]])) {

        new_df[[nm]] <- levels(model.frame(fit)[[nm]])[1]

      } else {

        new_df[[nm]] <- 0

      }

    }

    dX <- delta_design(fit, new_df, ref_df)

    # linear predictor difference

    lp <- as.numeric(dX %*% beta)

    # variance via delta method

    var_lp <- as.numeric(dX %*% V %*% t(dX))

    se <- sqrt(pmax(var_lp, 0))

    tibble::tibble(

      x = x,

      or = exp(lp),

      lo = exp(lp - 1.96 * se),

      hi = exp(lp + 1.96 * se)

    )

  })

  res

}

# Fit models and prepare plotting data for a given variable

fit_rcs_for_var <- function(data, varname, multivariable = FALSE, df_spline = 4) {

  # columns

  cols <- c("pre_ecl", varname, if (multivariable) covariates else character(0))

  d <- data[, cols, drop = FALSE] %>% tidyr::drop_na()

  d$pre_ecl <- as.integer(d$pre_ecl > 0)

  # formula strings

  rcs_term <- paste0("ns(`", varname, "`, df = ", df_spline, ")")

  if (multivariable && nchar(cov_terms) > 0) {

    full_formula   <- as.formula(paste0("pre_ecl ~ ", rcs_term, " + ", cov_terms))

    linear_formula <- as.formula(paste0("pre_ecl ~ `", varname, "` + ", cov_terms))

    base_formula   <- as.formula(paste0("pre_ecl ~ ", cov_terms))

  } else {

    full_formula   <- as.formula(paste0("pre_ecl ~ ", rcs_term))

    linear_formula <- as.formula(paste0("pre_ecl ~ `", varname, "`"))

    base_formula   <- as.formula("pre_ecl ~ 1")

  }

  # fit models

  full_fit   <- glm(full_formula,   data = d, family = binomial())

  linear_fit <- glm(linear_formula, data = d, family = binomial())

  base_fit   <- glm(base_formula,   data = d, family = binomial())

  # Likelihood-ratio tests

  lr_test <- function(f1, f0, df_diff) {

    LR <- 2 * (logLik(f1) - logLik(f0))

    p  <- pchisq(LR, df = df_diff, lower.tail = FALSE)

    as.numeric(p)

  }

  # df differences (at least 1)

  df_overall <- max(attr(logLik(full_fit), "df") - attr(logLik(base_fit), "df"), 1)

  df_nl      <- max(attr(logLik(full_fit), "df") - attr(logLik(linear_fit), "df"), 1)

  p_overall <- lr_test(full_fit, base_fit, df_overall)

  p_nl      <- lr_test(full_fit, linear_fit, df_nl)

  # grid in [1%, 99%]

  x <- as.numeric(d[[varname]])

  q <- stats::quantile(x, probs = c(0.01, 0.99), na.rm = TRUE)

  x_grid <- seq(q[1], q[2], length.out = 300)

  # reference value

  ref_val <- if (q[1] <= 0 && 0 <= q[2]) 0 else stats::median(x, na.rm = TRUE)

  # typical covariate values (for multivariable predictions)

  fixed_vals <- if (multivariable && length(covariates) > 0) {

    typical_values(d, covariates, cat_covs)

  } else {

    NULL

  }

  curve <- or_curve(full_fit, varname, x_grid, ref_val, fixed_vals)

  list(

    data = d,

    fit = full_fit,

    p_overall = p_overall,

    p_nl = p_nl,

    ref_val = ref_val,

    x_min = min(x_grid),

    x_max = max(x_grid),

    curve = curve

  )

}

fmt_p <- function(p) ifelse(is.na(p), "NA", ifelse(p < 0.001, "<0.001", sprintf("%.3f", p)))

# ---- 4) Fit four models

res_ft4_uni   <- fit_rcs_for_var(df, "FT4_change", multivariable = FALSE, df_spline = 4)

res_ft4_multi <- fit_rcs_for_var(df, "FT4_change", multivariable = TRUE,  df_spline = 4)

res_tsh_uni   <- fit_rcs_for_var(df, "TSH_change", multivariable = FALSE, df_spline = 4)

res_tsh_multi <- fit_rcs_for_var(df, "TSH_change", multivariable = TRUE,  df_spline = 4)

# ---- 5) Harmonize ranges and prepare plotting frames

# Shared x-range within each variable row

ft4_xlim <- c(min(res_ft4_uni$x_min, res_ft4_multi$x_min),

              max(res_ft4_uni$x_max, res_ft4_multi$x_max))

tsh_xlim <- c(min(res_tsh_uni$x_min, res_tsh_multi$x_min),

              max(res_tsh_uni$x_max, res_tsh_multi$x_max))

# Shared y-limit across all panels (linear)

all_hi <- max(res_ft4_uni$curve$hi, res_ft4_multi$curve$hi,

              res_tsh_uni$curve$hi, res_tsh_multi$curve$hi, na.rm = TRUE)

ymax <- ceiling(max(2, all_hi))

# Build a function to generate a panel dataframe with aesthetics

panel_df <- function(res, var_label, model_label) {

  res$curve %>%

    mutate(

      var = var_label,

      model = model_label,

      p_overall_str   = paste0("P overall = ", fmt_p(res$p_overall)),

      p_nl_str        = paste0("P nonlinear = ", fmt_p(res$p_nl)),

      ref_val = res$ref_val

    )

}

df_plot <- bind_rows(

  panel_df(res_ft4_uni,   "ΔFT4", "Univariable logistic regression with RCS"),

  panel_df(res_ft4_multi, "ΔFT4", "Multivariable logistic regression with RCS"),

  panel_df(res_tsh_uni,   "ΔTSH", "Univariable logistic regression with RCS"),

  panel_df(res_tsh_multi, "ΔTSH", "Multivariable logistic regression with RCS")

)

# Factor ordering for facetting

df_plot$model <- factor(df_plot$model,

                        levels = c("Univariable logistic regression with RCS",

                                   "Multivariable logistic regression with RCS"))

df_plot$var <- factor(df_plot$var, levels = c("ΔFT4", "ΔTSH"))

# Define color palette (blue for univariable, red for multivariable)

col_map <- c("Univariable logistic regression with RCS" = "#1f77b4",

             "Multivariable logistic regression with RCS" = "#d62728")

# Reference lines per facet: y = 1 and x = ref_val

# We'll add them inside geom layers using data trick per facet

ref_lines <- df_plot %>%

  group_by(var, model) %>%

  summarize(ref_val = unique(ref_val)[1], .groups = "drop")

# ---- 6) Plot (four panels, square, linear y, integer ticks)

p <- ggplot(df_plot, aes(x = x, y = or, group = model, color = model, fill = model)) +

  geom_ribbon(aes(ymin = lo, ymax = hi), alpha = 0.18, color = NA) +

  geom_line(size = 1.1) +

  # Horizontal OR = 1

  geom_hline(yintercept = 1, linetype = "dashed", color = "grey40", linewidth = 0.6) +

  # Vertical reference per panel

  geom_vline(data = ref_lines, aes(xintercept = ref_val),

             linetype = "dotted", color = "grey40", linewidth = 0.6) +

  scale_color_manual(values = col_map, guide = "none") +

  scale_fill_manual(values = col_map, guide = "none") +

  facet_grid(rows = vars(var), cols = vars(model), scales = "free_x") +

  labs(x = NULL, y = "OR (95% CI)") +

  # Linear y with integer ticks

  scale_y_continuous(limits = c(0, ymax), breaks = seq(0, ymax, by = 1), expand = c(0, 0)) +

  # Apply harmonized x-limits per row using coord_cartesian inside facetting

  # We'll do it by annotating after building the plot

  theme_bw(base_size = 12) +

  theme(

    strip.text = element_text(face = "bold"),

    panel.grid.major.y = element_line(linetype = "dotted", color = "grey80"),

    panel.grid.minor = element_blank(),

    # Square plotting area for each panel

    aspect.ratio = 1,

    axis.title.x = element_text(margin = margin(t = 6)),

    axis.title.y = element_text(margin = margin(r = 6))

  )

# Because facet_grid uses free_x, set x-limits per row via ggplot_build patch

# Simpler: split data into two grobs; here we can set after printing using coord_cartesian per row is non-trivial.

# Instead, we ensure df_plot already restricts x per row by dropping values outside desired xlim:

df_plot <- df_plot %>%

  group_by(var) %>%

  mutate(

    x_min_row = ifelse(var == "ΔFT4", ft4_xlim[1], tsh_xlim[1]),

    x_max_row = ifelse(var == "ΔFT4", ft4_xlim[2], tsh_xlim[2])

  ) %>%

  ungroup() %>%

  filter(x >= x_min_row, x <= x_max_row)

# Re-plot with trimmed data

p <- ggplot(df_plot, aes(x = x, y = or, group = model, color = model, fill = model)) +

  geom_ribbon(aes(ymin = lo, ymax = hi), alpha = 0.18, color = NA) +

  geom_line(size = 1.1) +

  geom_hline(yintercept = 1, linetype = "dashed", color = "grey40", linewidth = 0.6) +

  geom_vline(data = ref_lines, aes(xintercept = ref_val),

             linetype = "dotted", color = "grey40", linewidth = 0.6) +

  scale_color_manual(values = col_map, guide = "none") +

  scale_fill_manual(values = col_map, guide = "none") +

  facet_grid(rows = vars(var), cols = vars(model), scales = "free_x") +

  labs(x = NULL, y = "OR (95% CI)") +

  scale_y_continuous(limits = c(0, ymax), breaks = seq(0, ymax, by = 1), expand = c(0, 0)) +

  theme_bw(base_size = 12) +

  theme(

    strip.text = element_text(face = "bold"),

    panel.grid.major.y = element_line(linetype = "dotted", color = "grey80"),

    panel.grid.minor = element_blank(),

    aspect.ratio = 1,              # square panels (equal physical lengths of x and y)

    axis.title.x = element_text(margin = margin(t = 6)),

    axis.title.y = element_text(margin = margin(r = 6))

  ) +

  # axis labels per row

  labs(x = NULL) +

  # Add x-axis titles per row using strip labels: we will set them via annotate

  NULL

# Add x-axis labels by row using patchwork-like annotation:

# Simpler: print two separate plots stacked; but here we can set overall x labels with labs and use facet strips as titles.

# Instead, we set axis titles after drawing:

# Set x-axis titles directly in the grob is complex; easiest: add them as x-axis label globally.

# We'll add them manually via grid if needed; otherwise keep default axis titles below.

p <- p + labs(x = "")

# Print to device

print(p)

# ---- 7) Save files

ggsave("RCS_logit_linear.png", p, width = 12, height = 12, dpi = 300)
