# Supplementary material for: Maternal TSH and FT4 changes during pregnancy as risk factors for preeclampsia in euthyroid women
Source: Front Endocrinol (Lausanne). 2026 May 26;17:1782499. doi: 10.3389/fendo.2026.1782499 (PMC13246337; doi:10.3389/fendo.2026.1782499)
Supplement: Supplementary file 2 [file Table1.docx]

**Table S1. Standardized Mean Differences (SMDs) for Baseline Variables Across Thyroid Trajectory Groups**

| Variable | Category | SMD | | |
| --- | --- | --- | --- | --- |
|  |  | **Group 2 vs 1** | **Group 3 vs 1** | **Group 4 vs 1** |
| Parity | Nulliparous | 0.211 | 0.059 | 0.246 |
| Education | Bachelor’s degree or above | -0.046 | -0.053 | -0.065 |
| IVF conception | Yes | 0.013 | 0.042 | -0.001 |
| GDM | Yes | -0.081 | -0.017 | -0.047 |
| TPOAb | Positive | -0.094 | -0.219 | -0.240 |
| Smoking | Yes | 0.002 | -0.012 | -0.017 |
| Alcohol consumption | Yes | -0.017 | 0.007 | -0.017 |
| Age (years) | | -0.154 | -0.057 | -0.159 |
| Gestational age at sampling (weeks) | | 0.042 | -0.057 | -0.008 |
| Baseline early-pregnancy FT4 (pmol/L) | | 1.338 | 0.163 | 1.580 |
| Baseline early-pregnancy TSH (mIU/L) | | -0.238 | -0.691 | -1.043 |
| Pre-pregnancy BMI (kg/m²) | | -0.129 | -0.067 | -0.175 |

*SMD = Standardized Mean Difference, calculated between Group 1 (reference) and Groups 2–4.*
