# Supplementary material for: Maternal TSH and FT4 changes during pregnancy as risk factors for preeclampsia in euthyroid women
Source: Front Endocrinol (Lausanne). 2026 May 26;17:1782499. doi: 10.3389/fendo.2026.1782499 (PMC13246337; doi:10.3389/fendo.2026.1782499)
Supplement: Supplementary file 3 [file Table2.docx]

**Table S2. Sensitivity Analysis Based on TSH and FT4 Tertile-Based Classification**

| New Group | Definition | | Corresponding Original Group | Univariate Analysis | | Multivariate Analysis | |
| --- | --- | --- | --- | --- | --- | --- | --- |
|  | TSH change  （mIU/L） | FT4 change  （pmol/L） |  | OR (95% CI) | P | aOR (95% CI) | P |
| Group 1 | ≤ 0.68 | > -2.40 | Group 1 | 1.00 (Reference) | | 1.00 (Reference) | |
| Group 2 | > 0.68 | > -2.40 | Group 2 | 1.09 (0.83–1.44) | 0.536 | 1.08 (0.77–1.53) | 0.650 |
| Group 3 | ≤ 0.68 | -4.30 ~ -2.40 | Groups 1 + 3 | 1.16 (0.93–1.46) | 0.197 | 1.38 (1.05–1.81) | 0.019* |
| Group 4 | > 0.68 | -4.30 ~ -2.40 | Groups 2 + 4 | 1.34 (0.98–1.84) | 0.067 | 1.38 (0.94–2.04) | 0.102 |
| Group 5 | ≤ 0.68 | ≤ -4.30 | Group 3 | 1.53 (1.24–1.90) | <0.001* | 1.60 (1.22–2.11) | <0.001* |
| Group 6 | > 0.68 | ≤ -4.30 | Group 4 | 2.32 (1.82–2.96) | <0.001* | 2.40 (1.75–3.30) | <0.001* |

***Note:*** *The multivariate model was adjusted for maternal age, pre-pregnancy BMI, parity, education level, IVF status, TPOAb status, smoking, alcohol consumption, and GDM.*
